# Supplementary material for: Reliable identification of protein-protein interactions by crosslinking mass spectrometry
Source: Nat Commun. 2021 Jun 11;12:3564. doi: 10.1038/s41467-021-23666-z (PMC8196013; doi:10.1038/s41467-021-23666-z)
Supplement: Supplementary file 1 — Supplementary Information [file 41467_2021_23666_MOESM1_ESM.pdf]

Supplementary Materials for

Reliable identification of protein-protein interactions by crosslinking mass spectrometry

Swantje Lenz[1]*, Ludwig R. Sinn[1]*, Francis O'Reilly[1]*, Lutz Fischer[1], Fritz Wegner[1], Juri Rappsilber[1,2]

[1] Bioanalytics, Institute of Biotechnology, Technische Universität Berlin, 13355 Berlin, Germany
[2] Wellcome Centre for Cell Biology, University of Edinburgh, Edinburgh EH9 3BF, UK

*These authors contributed equally.

Correspondence to: Juri.Rappsilber@tu-berlin.de

This file includes:

Supplementary Table 1
Supplementary Figures 1-10

Supplementary Table 1: Recent large scale crosslinking mass spectrometry studies and aspects of their FDR method

Sample	Search / FDR Software	Self- / heteromeric crosslinks separated for FDR	FDR level	threshold	reference
Murine synaptosomes	XlinkX / PD	no	CSM	2%	1
Saccharomyces cerevisiae nucleus	XlinkX / PD	no	CSM	1%	2
Saccharomyces cerevisiae mitochondria	pLink1	no	CSM	1%	3
Several previously published datasets	pLink2	yes	CSM	5%	4
Drosophila melanogaster embryo lysate	MeroX	yes	CSM	1%	5
Human cells	Comet / XLinkProphet	yes	Peptide pair	1%	6
Human cell lysate	MaxLinker	no	Peptide pair	1%	7
Human cell lysate	XlinkX / PD	no	Peptide pair	1%	8
Saccharomyces cerevisiae mitochondria	Kojak	yes	Peptide pair	2%	9
Human cell lysate	xiSEARCH / xiFDR	yes	Residue pair	5%	10
Human mitochondria	xiSEARCH / xiFDR	yes	Residue pair	5%	11
Mycoplasma pneumoniae cells	xiSEARCH / xiFDR	yes	Residue pair & PPI	5%	12

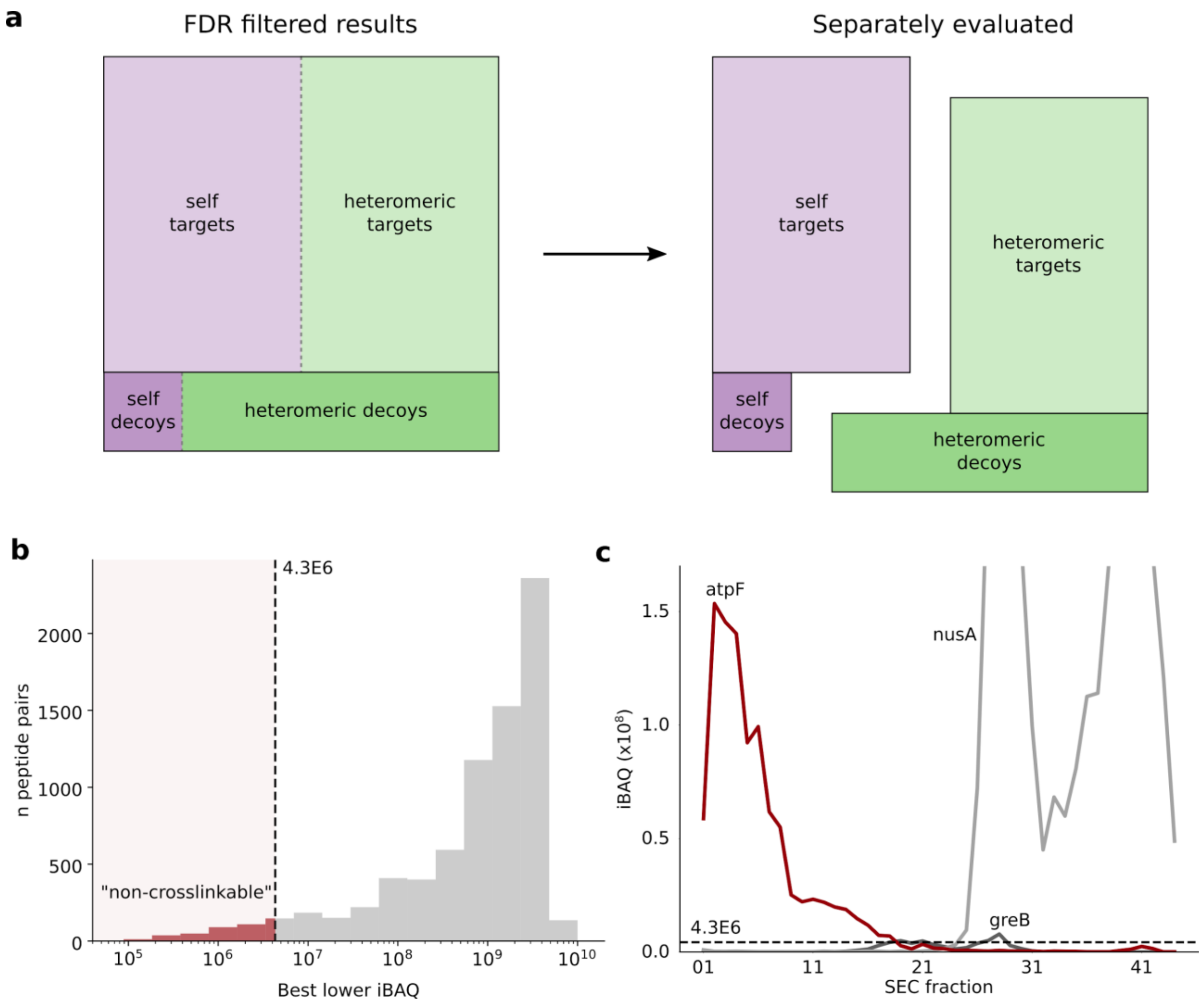

Supplementary Figure 1: Non-crosslinkable control definition.

a Illustration of the distribution of decoy matches for self and heteromeric crosslinks. If considered together for FDR calculation, heteromeric decoy matches will be matched more frequently and make up most of the summed decoy matches. If subsequently heteromeric matches are evaluated separately (e.g. for reporting PPIs), their error will be larger than the previously calculated FDR (which would only be correct for the data as a whole). **b** Abundance distribution of proteins identified with heteromeric crosslinks at a generous 10% heteromeric peptide pair FDR (best lower iBAQ, see Methods). Protein pairs are accepted as plausible if both proteins reach the 5th percentile in the same fraction, otherwise the pair is defined as 'non-crosslinkable'. 544,274 (6% of all possible PPIs in the *E. coli* proteome of 4350 proteins) PPIs are defined as plausible (Supplementary data 7), while 8,914,801 (94%) PPIs are non-crosslinkable. The dashed line represents the chosen 5th-percentile cutoff of 4.3E6. **c** Example of proteins defined either as plausible to crosslink to NusA (GreB, dark grey) or "non-crosslinkable" (AtpF, red). Although AtpF is present in some fractions together with NusA, it is too low abundant in those, therefore the proteins are defined as "non-crosslinkable". In contrast, GreB reaches the cutoff in the same fractions with NusA, therefore the two are crosslinkable. Source data are provided as a Source Data file.

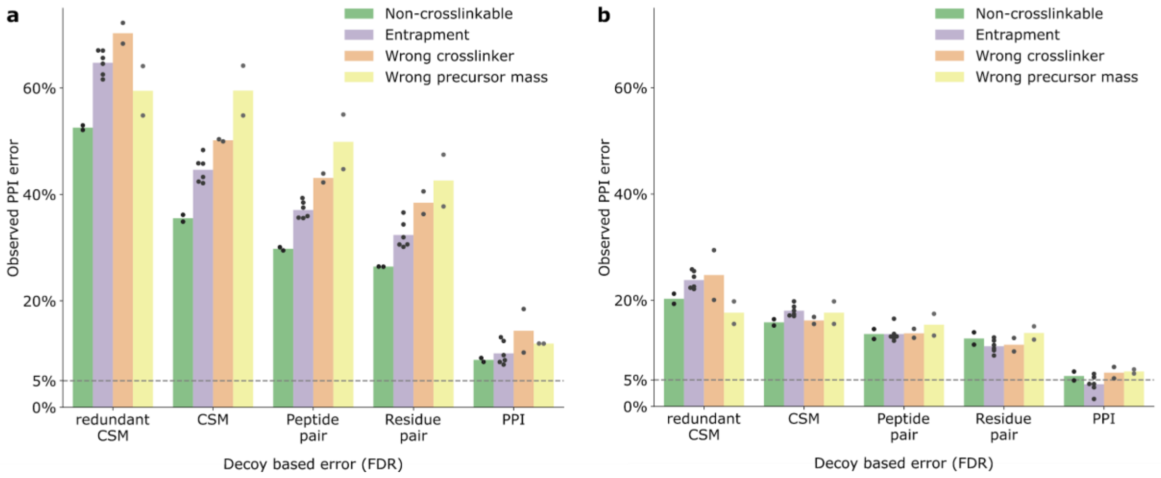

Supplementary Figure 2: Observed PPI-level error using a 5% FDR cutoff at different information levels applied to entrapment, wrong crosslinker, wrong precursor mass and non-crosslinkable controls.

Each bar represents a separate FDR calculation performed at different information levels on each of the controls. The y-axis displays the observed PPI-level errors of the respective controls employing a 5% **a** naive or **b** heteromeric FDR thresholds on different information levels. Bars represent the mean error, with individual data points shown on top (for each crosslinker dataset or entrapment database). Source data are provided as a Source Data file.

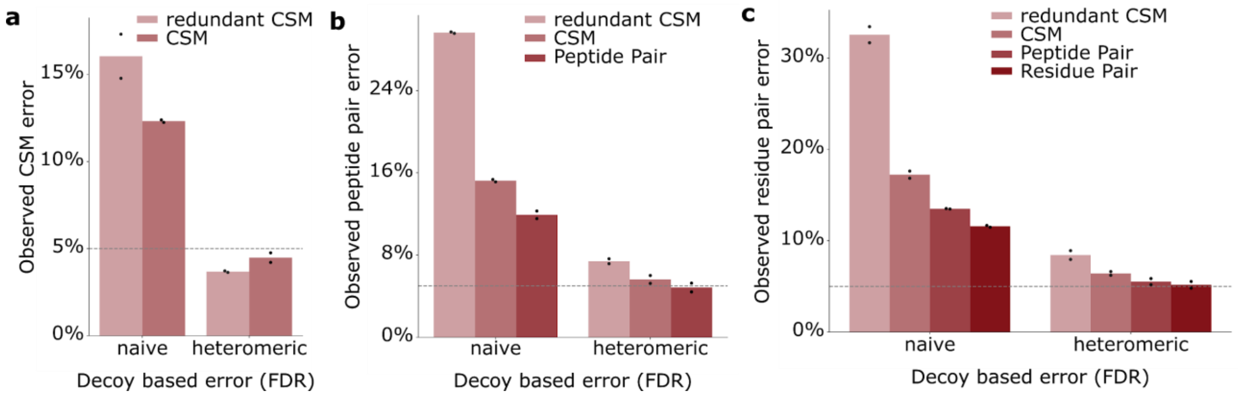

Supplementary Figure 3: **Observed errors using the non-crosslinkable control on information levels lower than PPI-level.**

Each bar represents a separate FDR calculation performed at different information levels while considering all CSMs (naive) or heteromeric crosslinks only (heteromeric). The y-axis displays the observed errors of the respective information level at 5% FDR. Observed error in **a** identified CSMs calculated on redundant or (unique) CSM-level, **b** identified peptide pairs calculated on redundant CSM-level, CSM-level and peptide pair-level, **c** identified residue pairs calculated on redundant CSM-level, CSM-level, peptide pair-level and residue pair-level, respectively. Individual values from DSSO and BS3 datasets are depicted by dots; the dashed line indicates the respective 5% FDR threshold. Source data are provided as a Source Data file.

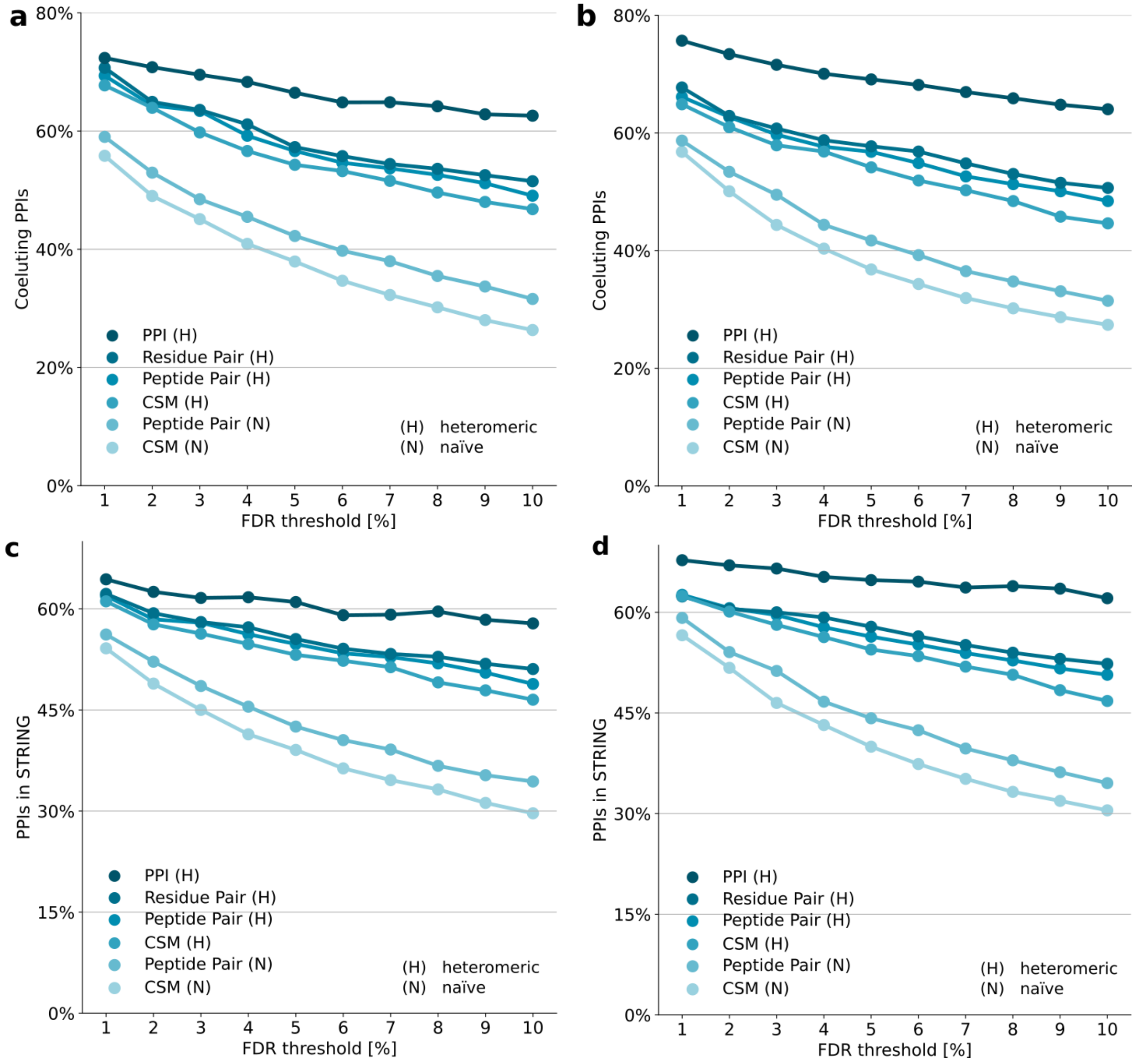

Supplementary Figure 4. Results of positive controls.

a, b Fraction of coeluting PPIs (correlation coefficient > 0.5) among the heteromeric PPIs passing a given FDR threshold, applying different published FDR approaches separated for **a** BS3 and **b** DSSO. **c**, **d** Fraction of PPIs present in the STRING database (STRING combined score >= 150) among the PPIs passing a given FDR threshold, applying different published FDR approaches (Supplementary Table 1) for **c** BS3 and **d** DSSO. Source data are provided as a Source Data file.

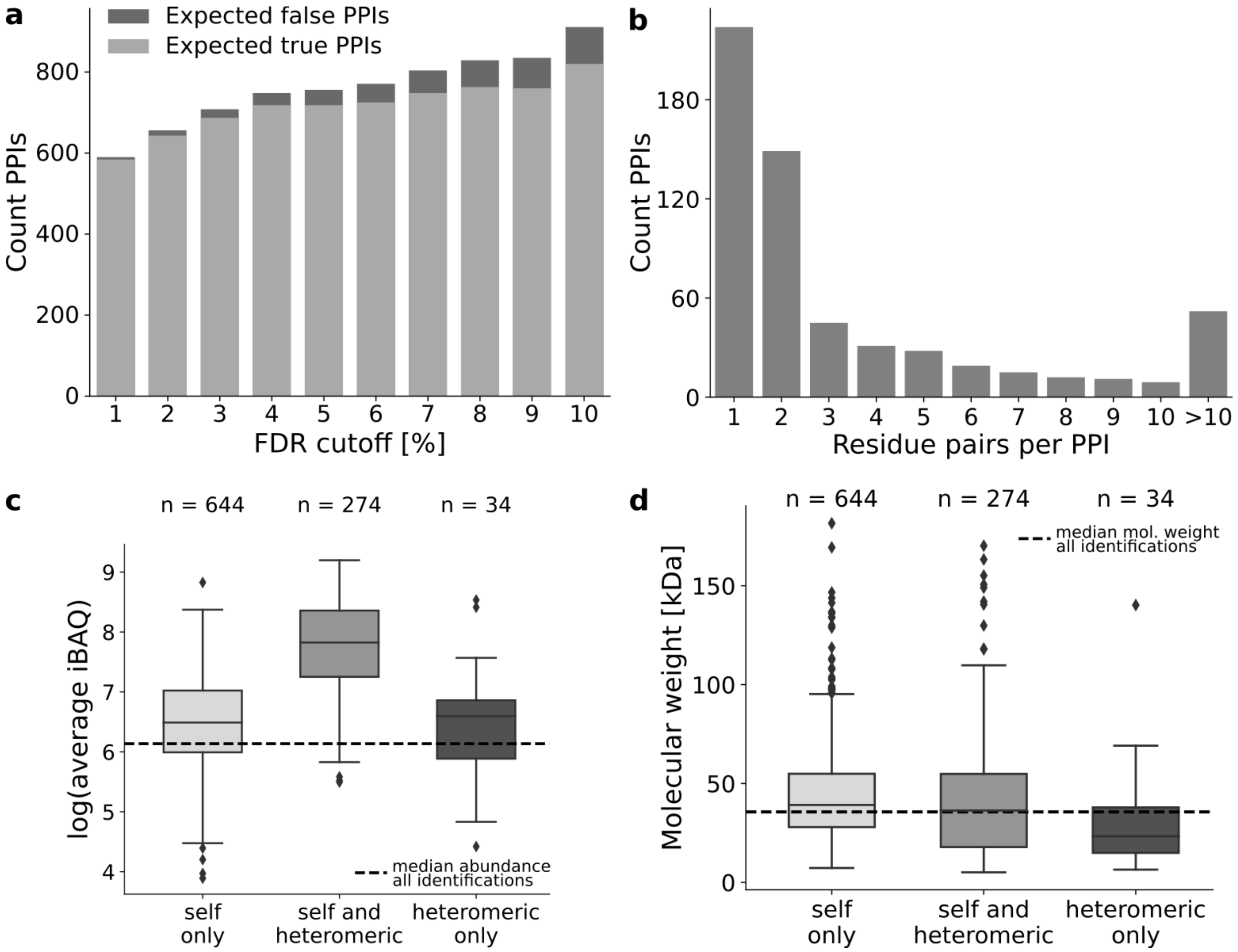

Supplementary Figure 5: Properties of final crosslink PPI network.

a The overall heteromeric PPI counts and the respective fraction of false PPIs are shown for varying FDR thresholds. Expected false PPIs were calculated based on decoy matches. For this dataset increasing FDR thresholds up to 4% add more true than false matches. **b** Distribution of residue pairs per PPI. **c** Abundance of identified crosslinked proteins in the respective categories. Heteromeric-only proteins are significantly more abundant than all identified proteins ($p = 0.044$ using a one-sided Kolmogorov–Smirnov test). **d** Heteromeric-only proteins tend to be smaller than the median of proteins in the database, and are therefore less likely to produce self-links. Boxplots in **c** and **d** depict the median (middle line), upper and lower quartiles (boxes), 1.5 times of the interquartile range (whiskers) as well as outliers (single points). Source data for panels c and d are provided as a Source Data file.

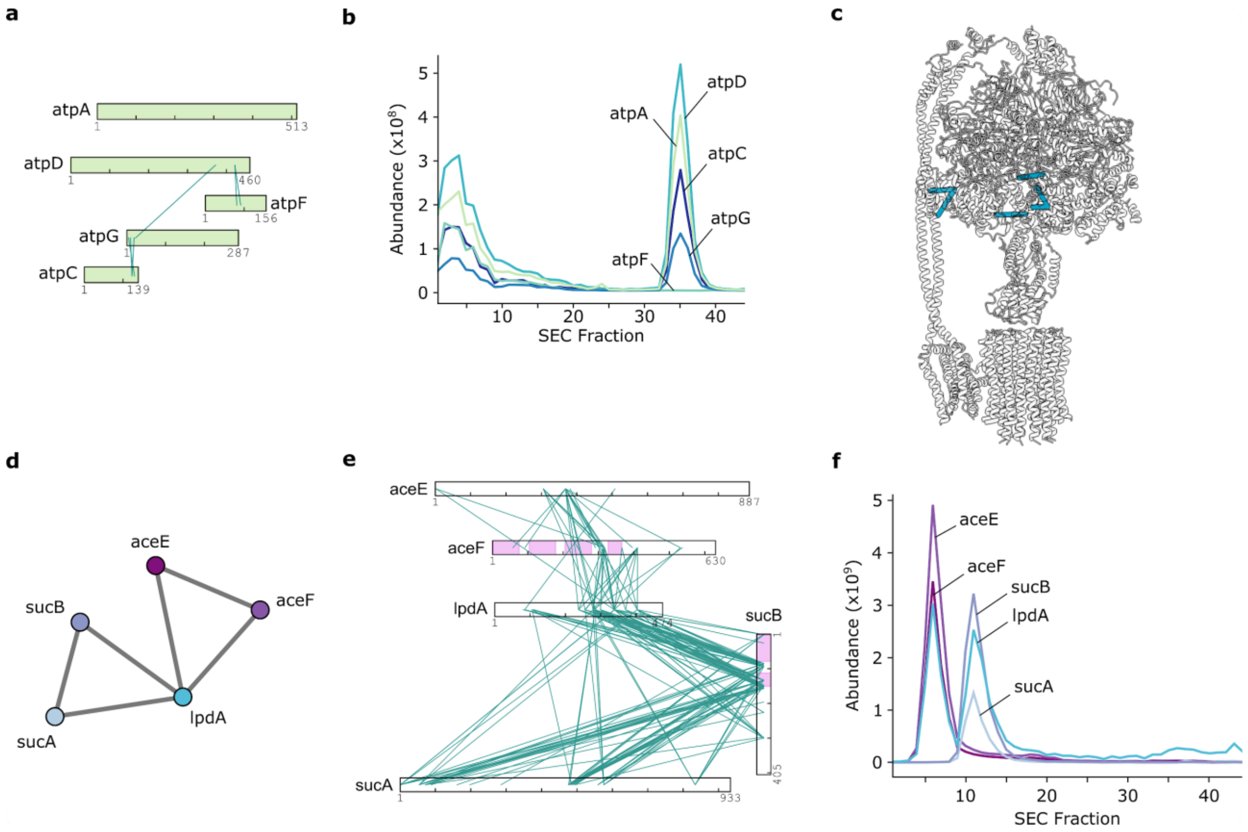

Supplementary Figure 6: ATP synthase and Pyruvate dehydrogenase Crosslinking MS subnetworks.

a PPI subnetwork of ATP synthase in xiNET[13]. Green shade on protein sequences illustrate sequence areas covered by the PDB model (PDB 5T4O)[14]. **b** SEC-Coelution traces of ATP synthase subunits. Note that all subunits also coelute in a very early fraction, probably containing lipid vesicles. **c** Structural model of ATP synthase with mapped heteromeric crosslinks (PDB 5T4O). All protein chains are colored in grey. Heteromeric links are below 35 Å and are colored in blue. **d** PPI subnetwork of pyruvate dehydrogenase and 2-oxoglutarate complexes with collapsed protein nodes and **e** with proteins shown as bars in xiNET[13]. **f** Coelution of pyruvate dehydrogenase / 2-oxoglutarate complex components showing lpdA eluting with both.

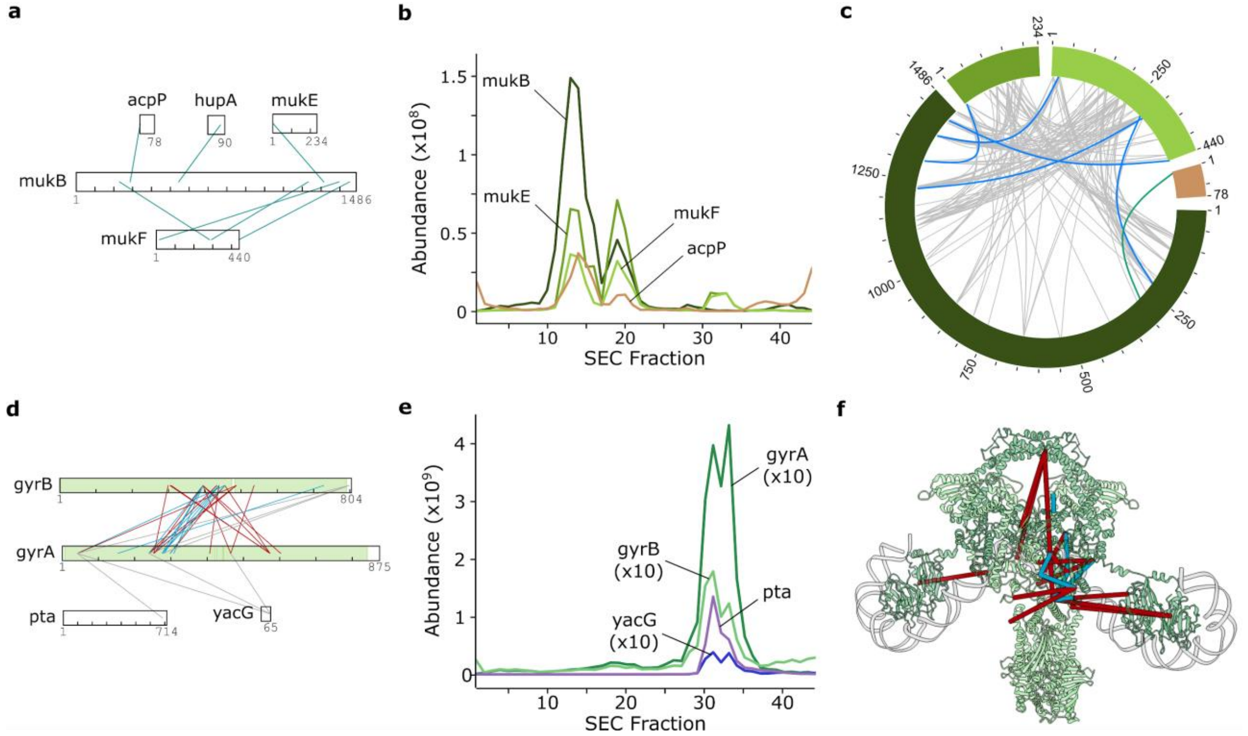

Supplementary Figure 7: MukBEF complex and DNA gyrase Crosslinking MS subnetworks.

a PPI subnetwork of MukBEF complex with proteins shown as bars in xiNET[13]. **b** Coelution traces of MukBEF complex with its binder AcpP (acpP). The MukBEF complex (fraction 13) dissociated into another MukBEF assembly with lower occupancy for MukB (fraction 19) as judged by coelution. The interactor AcpP is a known binder of MukBEF that could be confirmed by Crosslinking MS and coelution. **c** Crosslink distribution on MukBEF protein sequences compared to a recent *in-vitro* study[15]. Crosslinks are colored in blue if shared between the studies, in grey for *in-vitro* data only and in green if unique to this study. **d** PPI subnetwork of DNA gyrase in xiNET[13]. Green shade on protein sequences illustrate sequence areas covered by the PDB model (PDB 6RKW) [16]. Heteromeric links are colored in blue if below 35 Å, red when exceeding and grey when absent from the model used. **e** Coelution of DNA gyrase complex components with its inhibitor YacG and phosphate acetyltransferase Pta. The abundances for GyrA, GyrB and YacG were magnified 10-fold. **f** Structural model of DNA gyrase with mapped heteromeric crosslinks (PDB 6RKW) [16]. GyrA is shown in dark green and GyrB in light green. DNA is shown in grey. For crosslink coloration see panel **d**. Of note, the DNA gyrase inhibitor YacG was suggested to associate with proteins involved in coenzyme A metabolism such as Pta[17], which is supported by this analysis.

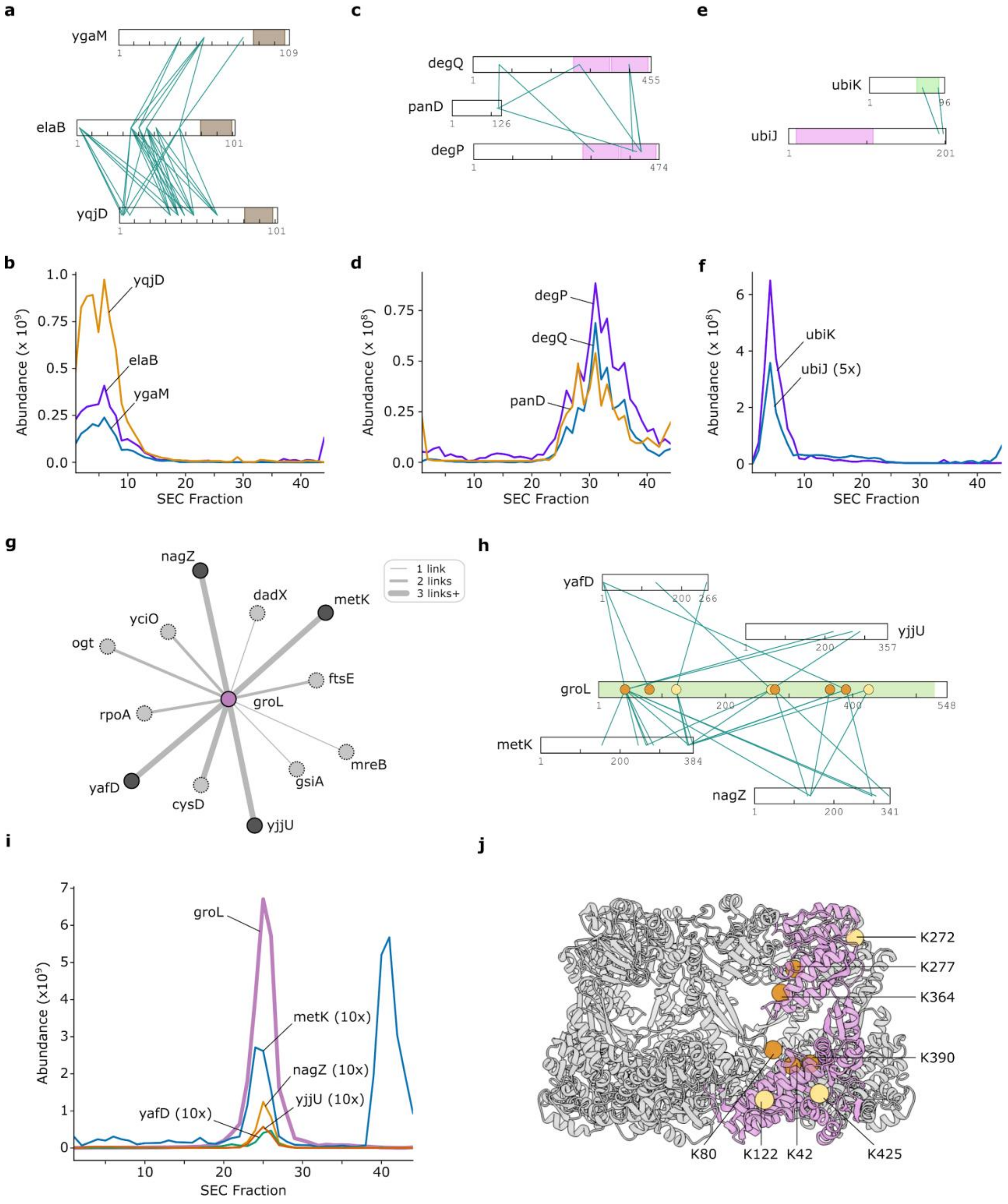

Supplementary Figure 8: Novel identified PPIs.

a, c, e, h Selected heteromeric PPI networks with proteins shown as bars or **g** in a network diagram[13] **b, d, f, i** with their corresponding SEC elution traces. Coloration in panels **a, c, e** represent: transmembrane domains for elaB network; PDZ domains for degP and degQ (pink); coiled-coil domain (green) for ubiK and SCP2 domain for ubiJ (pink). The abundance for ubiJ in

panel **f** was magnified 5-fold; abundances of GroEL interactors in panel **i** were magnified 10-fold. High-confidence binders of GroEL, displayed with darker nodes in panel **g**, were selected for the panels **h** & **i**. **h** Selected binders of GroEL shown as bars[13]. Green areas mark GroEL's aligned region with a structural model (PDB 4PKO)[18]. Lysines on the inside of GroEL are highlighted with an orange circle, the ones on the outside with a circle in light yellow. **i** SEC coelution trace of GroEL and selected binders. **j** GroEL structural model (PDB 4PKO) [18] with crosslinked residues highlighted. One ring of the GroEL barrel assembly is shown with one subunit of GroEL colored in pink. Crosslinked lysine residues are indicated as spheres and colored as in panel **h**.

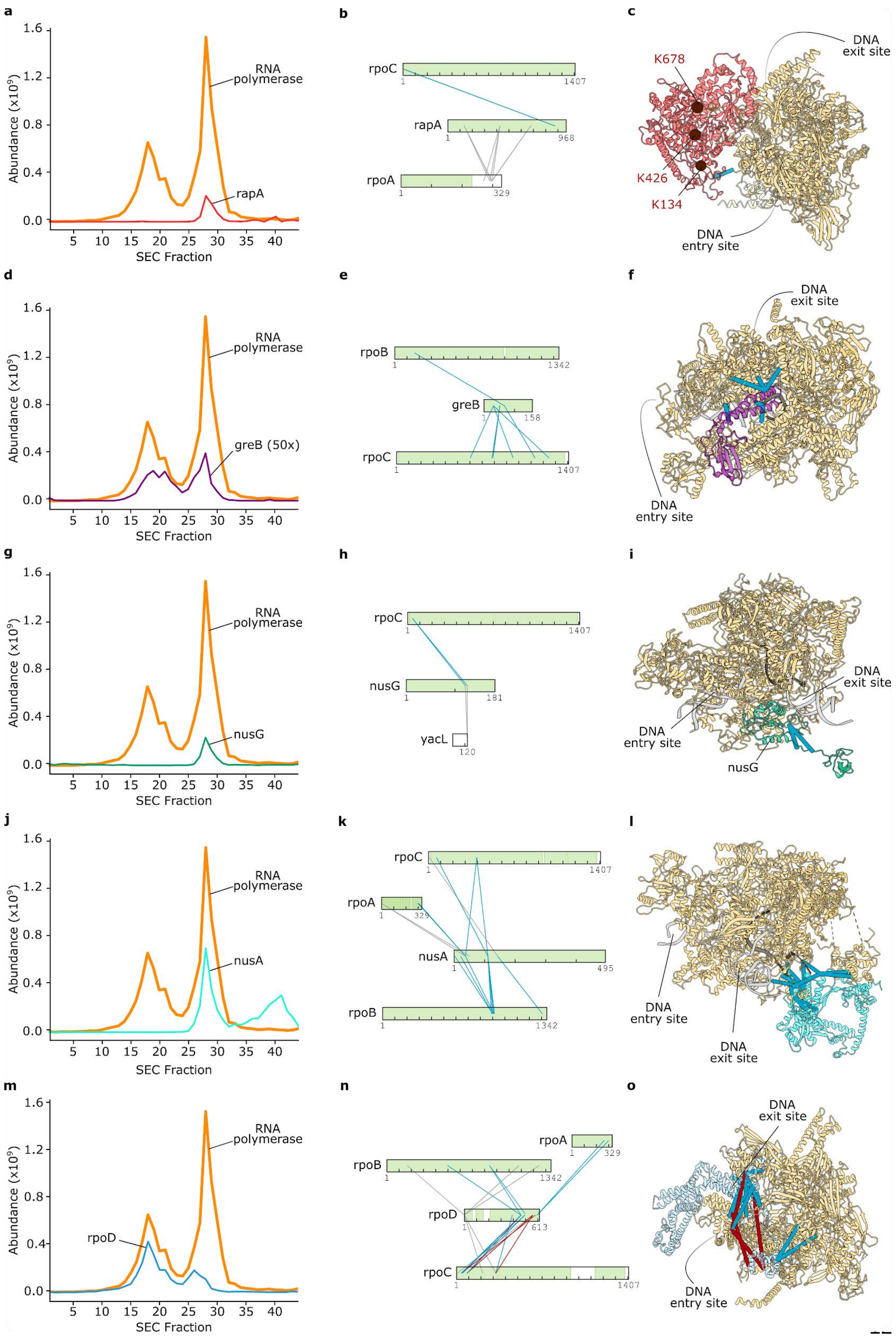
a
Abundance (x10^9)
SEC Fraction
RNA polymerase
rapA
b
rpoC
rapA
rpoA
c
K678
K426
K134
DNA exit site
DNA entry site
d
greB (50x)
e
rpoB
greB
rpoC
f
g
nusG
h
nusG
yacL
i
j
nusA
k
l
m
rpoD
n
rpoA
rpoB
rpoD
rpoC
o

Supplementary Figure 9: Selected RNA polymerase binders.

a, d, g, j, m Collected experimental support for heteromeric PPIs between RNAP and selected binders - rapA, greB, nusG, nusA and rpoD - is shown from SEC elution profiles, **b, e, h, k, n** Crosslinking MS-based PPI screening in xiNET[13] and **c, f, i, l, o** matching to existing structural PPI models. The abundance of greB was magnified 50-fold. Green shade on protein sequence bars illustrate areas covered by PDB models that were used to map heteromeric links onto structures. In protein crosslink diagrams and protein structural models, heteromeric crosslinks satisfying euclidean distance thresholds of 35 Å are shown in blue while longer links are colored in red. The structural models used were: rapA (PDB 4S20)[19], greB (PDB 6RIN) [20], nusG (PDB 5MS0) [21], nusA (PDB 6FLQ) [22] and rpoD (PDB 4ZH3) [23]. If present in the model, DNA is colored in grey and RNA in black. Labeled spheres in panel **c** mark lysine residues crosslinked to C-termini of RNAP's α-subunits (absent from this model).

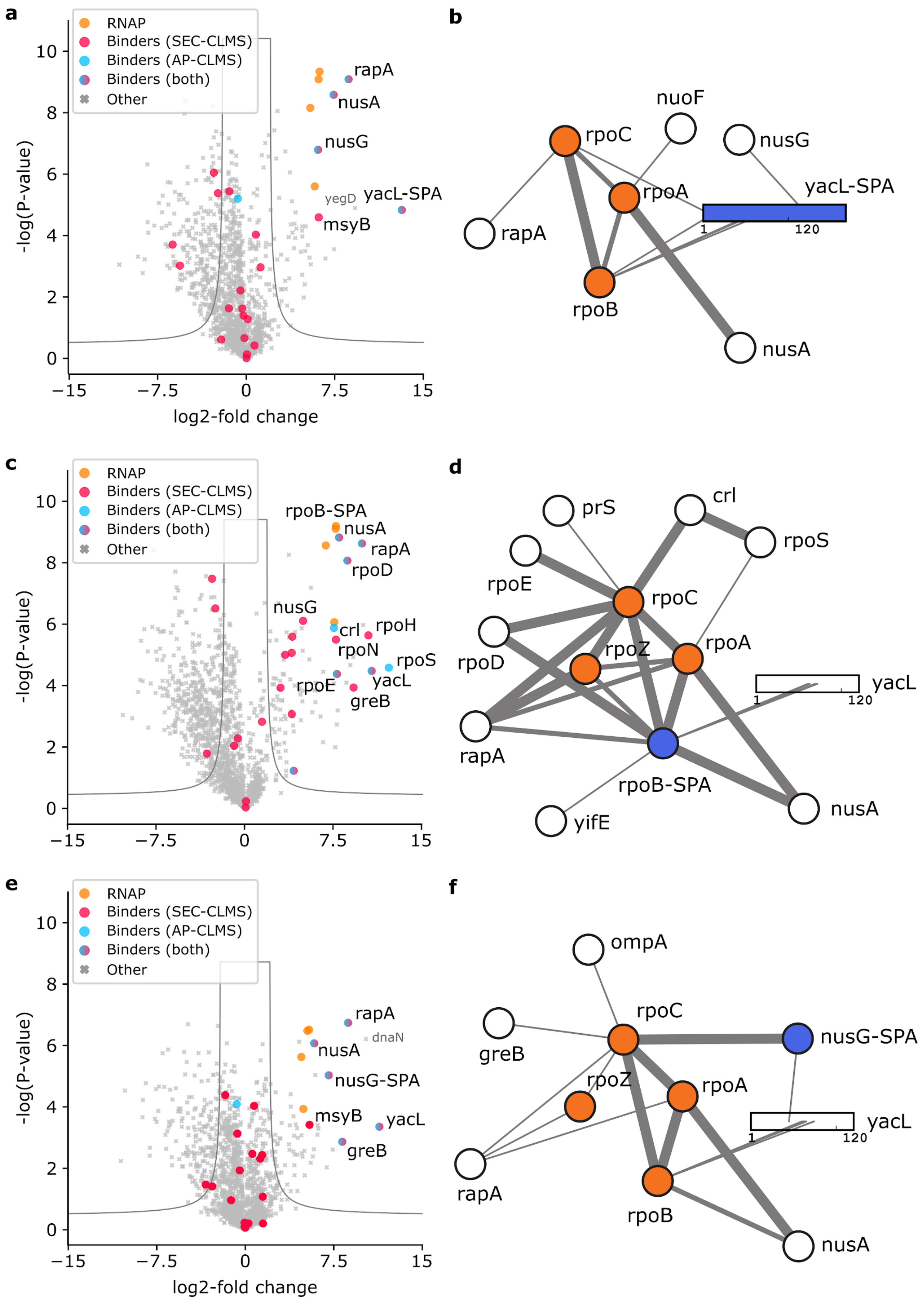
a
b
c
d
e
f
RNAP
Binders (SEC-CLMS)
Binders (AP-CLMS)
Binders (both)
Other
-log(P-value)
log2-fold change
rapA
nusA
nusG
yegD
yacL-SPA
msyB
nuoF
rpoC
rpoA
rpoB
rpoB-SPA
rpoD
rpoH
rpoN
rpoS
rpoE
crl
yacL
greB
prS
rpoZ
yifE
dnaN
nusG-SPA
ompA
120

Supplementary Figure 10: AP-MS and sulfo-SDA crosslinking.

Enrichment analysis from affinity-enriched **a** YacL-SPA, **c** RpoB-SPA and **e** NusG-SPA with **b, d, f** corresponding crosslink subnetworks from sulfo-SDA crosslinked eluates from these enrichments. In the volcano plots, proteins of interest are labelled as follows: components of RNA polymerase in orange; proteins found crosslinked to RNA polymerase from lysate SEC fractionation in red; proteins found crosslinked to RNA polymerase from a given affinity-enrichment experiment in blue; overlapping proteins between the two sample workflows in red and blue; all other proteins are indicated as grey crosses. In the crosslink subnetworks generated with xiNET[13], RNA polymerase constituents are labelled in orange and SPA-tagged proteins are highlighted in blue. YacL is shown in expanded sequence view. The thickness of the lines represents the number of unique residue pairs between the proteins.

Supplementary References

1. Gonzalez-Lozano, M. A. *et al.* Stitching the synapse: Cross-linking mass spectrometry into resolving synaptic protein interactions. *Sci Adv* **6**, eaax5783 (2020).
2. Bartolec, T. K. et al. Cross-linking Mass Spectrometry Analysis of the Yeast Nucleus Reveals Extensive Protein-Protein Interactions Not Detected by Systematic Two-Hybrid or Affinity Purification-Mass Spectrometry. Anal. Chem. 92, 1874–1882 (2020).
3. Linden, A. *et al.* A cross-linking mass spectrometry approach defines protein interactions in yeast mitochondria. *Mol. Cell. Proteomics* (2020) doi:10.1074/mcp.RA120.002028.
4. Chen, Z.-L. *et al.* A high-speed search engine pLink 2 with systematic evaluation for proteome scale identification of cross-linked peptides. *Nat. Commun.* **10**, 3404 (2019).
5. Götze, M., Iacobucci, C., Ihling, C. H. & Sinz, A. A Simple Cross-Linking/Mass Spectrometry Workflow for Studying System-wide Protein Interactions. Anal. Chem. 91, 10236–10244 (2019).
6. Chavez, J. D., Keller, A., Zhou, B., Tian, R. & Bruce, J. E. Cellular Interactome Dynamics during Paclitaxel Treatment. *Cell Rep.* **29**, 2371–2383.e5 (2019).
7. Yugandhar, K. *et al.* MaXLinker: Proteome-wide Cross-link Identifications with High Specificity and Sensitivity. *Mol. Cell. Proteomics* **19**, 554–568 (2020).
8. Steigenberger, B., Pieters, R. J., Heck, A. J. R. & Scheltema, R. A. PhoX: An IMAC-Enrichable Cross-Linking Reagent. *ACS Cent Sci* **5**, 1514–1522 (2019).
9. Makepeace, K. A. T. *et al.* Improving Identification of In-organello Protein-Protein Interactions Using an Affinity-enrichable, Isotopically Coded, and Mass Spectrometry-cleavable Chemical Crosslinker. *Mol. Cell. Proteomics* **19**, 624–639 (2020).
10. Mendes, M. L. *et al.* An integrated workflow for crosslinking mass spectrometry. *Mol. Syst. Biol.* **15**, e8994 (2019).
11. Ryl, P. S. J. et al. In Situ Structural Restraints from Crosslinking Mass Spectrometry in

Human Mitochondria. J. Proteome Res. (2019) doi:10.1021/acs.jproteome.9b00541.

12. O'Reilly, F. J. *et al.* In-cell architecture of an actively transcribing-translating expressome. *Science* **369**, 554–557 (2020).
13. Combe, C. W., Fischer, L. & Rappsilber, J. xiNET: cross-link network maps with residue resolution. *Mol. Cell. Proteomics* 14, 1137–1147 (2015).
14. Sobti, M. *et al.* Cryo-EM structures of the autoinhibited E. coli ATP synthase in three rotational states. *Elife* **5**, (2016).
15. Bürmann, F. *et al*. A folded conformation of MukBEF and cohesin. *Nat. Struct. Mol. Biol.* **26**, 227–236 (2019).
16. Vanden Broeck, A., Lotz, C., Ortiz, J. & Lamour, V. Cryo-EM structure of the complete E. coli DNA gyrase nucleoprotein complex. *Nat. Commun.* **10**, 4935 (2019).
17. Vos, S. M. *et al*. Direct control of type IIA topoisomerase activity by a chromosomally encoded regulatory protein. *Genes Dev*. **28**, 1485-1497 (2014).
18. Fei, X., Ye, X., LaRonde, N. A. & Lorimer, G. H. Formation and structures of GroEL:GroES2 chaperonin footballs, the protein-folding functional form. *Proc. Natl. Acad. Sci. U. S. A.* **111**, 12775–12780 (2014).
19. Liu, B., Zuo, Y. & Steitz, T. A. Structural basis for transcription reactivation by RapA. *Proc. Natl. Acad. Sci. U. S. A.* **112**, 2006–2010 (2015).Asda
20. Abdelkareem, M. 'men *et al.* Structural Basis of Transcription: RNA Polymerase Backtracking and Its Reactivation. *Mol. Cell* **75**, 298–309.e4 (2019).
21. Said, N. *et al.* Structural basis for λN-dependent processive transcription antitermination. *Nat Microbiol* **2**, 17062 (2017).
22. Guo, X. *et al.* Structural Basis for NusA Stabilized Transcriptional Pausing. *Mol. Cell* **69**, 816–827.e4 (2018).
23. Feng, Y. *et al.* Structural Basis of Transcription Inhibition by CBR Hydroxamidines and CBR Pyrazoles. *Structure* **23**, 1470–1481 (2015).
