# Supplementary material for: Reliable identification of protein-protein interactions by crosslinking mass spectrometry
Source: Nat Commun. 2021 Jun 11;12:3564. doi: 10.1038/s41467-021-23666-z (PMC8196013; doi:10.1038/s41467-021-23666-z)
Supplement: Supplementary file 3 — Description of Additional Supplementary Files [file 41467_2021_23666_MOESM3_ESM.docx]

Description of Additional Supplementary Files

**Supplementary Data 1**

**Description:** Detected heteromeric PPIs at 1% FDR

| **Column header** | **Explanation** |
| --- | --- |
| Protein1_UniProt_Accession | Uniprot Accession for Protein1 |
| Gene_name1 | primary Gene name for Protein1 |
| Protein1_UniProt_Accession | Uniprot Accession for Protein2 |
| Gene_name2 | primary Gene name for Protein2 |
| heteromeric_PPI-FDR | FDR value of the PPI from xiFDR |
| count_CSMs_total | total CSM count for a PPI |
| count_peptide_pairs_total | total peptide pair count for a PPI |
| count_residue_pairs_total | total residue pair count for a PPI |
| count_CSMs_DSSO | CSM count for a PPI from DSSO dataset |
| count_CSMs_BS3 | CSM count for a PPI from BS3 dataset |
| count_peptide_pairs_DSSO | unique peptide pair count for a PPI from DSSO dataset |
| count_peptide_pairs_BS3 | unique peptide pair count for a PPI from BS3 dataset |
| count_residue_pairs_DSSO | unique residue pair count for a PPI from DSSO dataset |
| count_residue_pairs_BS3 | unique residue pair count for a PPI from BS3 dataset |
| STRING_experimental_score | experimental score from STRING-database v10.5 |
| STRING_combined_score | combined score from STRING-database v10.5 |
| SEC_elution_correlation_coefficient | correlation coefficient of the two protein elution profiles, windowed |
| frac_left | first fraction of the elution window of a protein for correlation calculation |
| frac_right | last fraction of the elution window of a protein for correlation calculation |

**Supplementary Data 2**

**Description:** Protein quantitation from SEC fractionation

| **Column header** | **Explanation** |
| --- | --- |
| Protein_UniProt_Accession | Uniprot Accession for Protein (group) |
| Protein_names | Protein (group) name |
| Gene_names | primary Gene names (group) |
| Molecular_weight | Molecular weight of monomeric protein (group) |
| Total_iBAQ | Sum of protein abundance over all fractions |
| iBAQ_1 - 44 | Protein abundance in fractions 1 to 44 |

**Supplementary Data 3**

**Description:** Unique residue pairs detected in YacL-SPA affinity purification

| **Column header** | **Explanation** |
| --- | --- |
| Protein1_UniProt_Accession | Uniprot Accession for Protein1 |
| Protein2_UniProt_Accession | Uniprot Accession for Protein2 |
| Score | residue pair score |
| SeqPos1 | position of crosslinked residue 1 in Uniprot sequence |
| SeqPos2 | position of crosslinked residue 2 in Uniprot sequence |

**Supplementary Data 4**

**Description:** Unique residue pairs detected in RpoB-SPA affinity purification

| **Column header** | **Explanation** |
| --- | --- |
| Protein1_UniProt_Accession | Uniprot Accession for Protein1 |
| Protein2_UniProt_Accession | Uniprot Accession for Protein2 |
| Score | residue pair score |
| SeqPos1 | position of crosslinked residue 1 in Uniprot sequence |
| SeqPos2 | position of crosslinked residue 2 in Uniprot sequence |

**Supplementary Data 5**

**Description:** Unique residue pairs detected in NusG-SPA affinity purification

| **Column header** | **Explanation** |
| --- | --- |
| Protein1_UniProt_Accession | Uniprot Accession for Protein1 |
| Protein2_UniProt_Accession | Uniprot Accession for Protein2 |
| Score | residue pair score |
| SeqPos1 | position of crosslinked residue 1 in Uniprot sequence |
| SeqPos2 | position of crosslinked residue 2 in Uniprot sequence |

**Supplementary Data 6**

**Description:** PCR primers for SPA-tag strain validation

| **Column header** | **Explanation** |
| --- | --- |
| Gene region of interest | Gene region under study with this primer |
| Primer name | Name of the primer |
| Primer sequence (5' - 3') | Sequence of the primer (5' to 3') |
| Primer direction | forward or reverse primer |

**Supplementary Data 7**

**Description:** All plausible PPIs based on SEC fractionation

| **Column header** | **Explanation** |
| --- | --- |
| Protein1_UniProt_Accession | Uniprot Accession for Protein1 |
| Protein2_UniProt_Accession | Uniprot Accession for Protein2 |

**Supplementary Data 8**

**Description:** Self and heteromeric unique residue pairs of detected PPIs

| **Column header** | **Explanation** |
| --- | --- |
| Crosslinker | crosslinker(s) for which residue pair was identified |
| Protein1_UniProt_Accession | Uniprot Accession for Protein1 |
| Protein2_UniProt_Accession | Uniprot Accession for Protein2 |
| Score_BS3 | residue pair score in BS3 |
| Score_DSSO | residue pair score in DSSO |
| SeqPos1 | position of crosslinked residue 1 in Uniprot sequence |
| SeqPos2 | position of crosslinked residue 2 in Uniprot sequence |
