# Supplementary material for: Reliable identification of protein-protein interactions by crosslinking mass spectrometry
Source: Nat Commun. 2021 Jun 11;12:3564. doi: 10.1038/s41467-021-23666-z (PMC8196013; doi:10.1038/s41467-021-23666-z)
Supplement: Supplementary file 12 — Reporting Summary [file 41467_2021_23666_MOESM12_ESM.pdf]

nature research

Corresponding author(s): Juri Rappsilber

Last updated by author(s): Apr 27, 2021

Reporting Summary

Nature Research wishes to improve the reproducibility of the work that we publish. This form provides structure for consistency and transparency in reporting. For further information on Nature Research policies, see our Editorial Policies and the Editorial Policy Checklist.

Statistics

For all statistical analyses, confirm that the following items are present in the figure legend, table legend, main text, or Methods section.

n/a	Confirmed	
☐	☒	The exact sample size (*n*) for each experimental group/condition, given as a discrete number and unit of measurement
☐	☒	A statement on whether measurements were taken from distinct samples or whether the same sample was measured repeatedly
☐	☒	The statistical test(s) used AND whether they are one- or two-sided<br>*Only common tests should be described solely by name; describe more complex techniques in the Methods section.*
☒	☐	A description of all covariates tested
☒	☐	A description of any assumptions or corrections, such as tests of normality and adjustment for multiple comparisons
☐	☒	A full description of the statistical parameters including central tendency (e.g. means) or other basic estimates (e.g. regression coefficient) AND variation (e.g. standard deviation) or associated estimates of uncertainty (e.g. confidence intervals)
☐	☒	For null hypothesis testing, the test statistic (e.g. *F*, *t*, *r*) with confidence intervals, effect sizes, degrees of freedom and *P* value noted<br>*Give P values as exact values whenever suitable.*
☒	☐	For Bayesian analysis, information on the choice of priors and Markov chain Monte Carlo settings
☒	☐	For hierarchical and complex designs, identification of the appropriate level for tests and full reporting of outcomes
☐	☒	Estimates of effect sizes (e.g. Cohen's *d*, Pearson's *r*), indicating how they were calculated

Our web collection on statistics for biologists contains articles on many of the points above.

Software and code

Policy information about availability of computer code

Data collection	Tune 2.9 / 2.11 / 3.4, Xcalibur 4.1 / 4.2 / 4.4, SII for Xcalibur 1.4 / 1.5 / 1.6
Data analysis	MaxQuant 1.6.0.16, Perseus 1.5.6.0, msConvert 3.0.11729, xiSEARCH 1.6.746 / 1.7.6.2, xiFDR 2.0.dev / 2.1.5 (https://github.com/Rappsilber-Laboratory/xiFDR), python 3.7, seaborn 0.9.0, ChimeraX 0.92, DisVis 2.0, xiNET 1.0.0

For manuscripts utilizing custom algorithms or software that are central to the research but not yet described in published literature, software must be made available to editors and reviewers. We strongly encourage code deposition in a community repository (e.g. GitHub). See the Nature Research guidelines for submitting code & software for further information.

Data

Policy information about availability of data

All manuscripts must include a data availability statement. This statement should provide the following information, where applicable:

- Accession codes, unique identifiers, or web links for publicly available datasets
- A list of figures that have associated raw data
- A description of any restrictions on data availability

Raw data and MaxQuant outputs from quantitative proteomics SEC-MS experiments were deposited with the ProteomeXchange Consortium partner repository jPOSTrepo under the accession JPST000843 / PXD019004[https://repository.jpostdb.org/entry/JPST000843]56. Raw data and MaxQuant outputs from quantitative proteomics AP-MS experiments were deposited with the ProteomeXchange Consortium partner repository jPOSTrepo under the accession JPST001090 / PXD024146[https://repository.jpostdb.org/entry/JPST001090]56. All raw data, peak lists and search result files from BS3/DSSO crosslinking experiments in the SEC fractions and after multidimensional fractionation were deposited with the ProteomeXchange Consortium partner repository jPOSTrepo under the accession JPST000845 / PXD019120[https://repository.jpostdb.org/entry/JPST000845]56.
All raw data, peak lists and search result files from affinity-enrichment and crosslinking experiments were deposited with the ProteomeXchange Consortium partner

repository jPOSTrepo under the accession JPST001091 / PXD024148[https://repository.jpostdb.org/entry/JPST001091]56.
Source data for figures / panels 1b, 2a, 2b, 2d and Supplementary Figures 2, 3, 4, 5c, 5d are provided with this paper.
We accessed the STRING database (v10.5) via https://string-db.org/. The new link for this version is https://version-10-5.string-db.org/. The used resource can be downloaded using the following link: https://version-10-5.string-db.org/download/protein.links.detailed.v10.5/511145.protein.links.detailed.v10.5.txt.gz.

Models from the protein data bank (PDB) can be found under the following links:
http://doi.org/10.2210/pdb5T4O/pdb, http://doi.org/10.2210/pdb6RKW/pdb, http://doi.org/10.2210/pdb4PKO/pdb, http://doi.org/10.2210/pdb4S20/pdb, http://doi.org/10.2210/pdb6RIN/pdb, http://doi.org/10.2210/pdb5MS0/pdb, http://doi.org/10.2210/pdb6FLQ/pdb, http://doi.org/10.2210/pdb4ZH3/pdb, http://doi.org/10.2210/pdb6c6u/pdb.

Field-specific reporting

Please select the one below that is the best fit for your research. If you are not sure, read the appropriate sections before making your selection.

[x] Life sciences [ ] Behavioural & social sciences [ ] Ecological, evolutionary & environmental sciences

For a reference copy of the document with all sections, see nature.com/documents/nr-reporting-summary-flat.pdf

Life sciences study design

All studies must disclose on these points even when the disclosure is negative.

Sample size

The sample size was not predetermined. We strived to generate a large dataset containing thousands of cross-linked spectrum matches by using two different crosslinkers. This led to hundreds of detected protein-protein-interactions that we leveraged to test multiple error estimation approaches. Since the outcome from using different error estimation approaches resulted in a reasonably fine-grained picture when comparing these approaches, we reason this sample size sufficient.
As pointed out by one reviewer in addition, the here presented crosslink datasets exceed the typically rather small crosslinking mass spectrometry datasets.

Data exclusions

No data were excluded.

Replication

The independent datasets from using the BS3 and DSSO crosslinkers were each acquired once and treated as replicates. All attempts at replication were successful.

Randomization

SEC-MS protein profiles were acquired in logical sequences (i.e. adjacent SEC fractions) to minimize the influence of sample carry-over within the acquisition regime.
Crosslink mass spectrometry acquisitions were not randomized.

Blinding

Blinding was not relevant to this study as there was no observer bias to be expected.

Reporting for specific materials, systems and methods

We require information from authors about some types of materials, experimental systems and methods used in many studies. Here, indicate whether each material, system or method listed is relevant to your study. If you are not sure if a list item applies to your research, read the appropriate section before selecting a response.

Materials & experimental systems

n/a	Involved in the study
[x]	[ ] Antibodies
[x]	[ ] Eukaryotic cell lines
[x]	[ ] Palaeontology and archaeology
[x]	[ ] Animals and other organisms
[x]	[ ] Human research participants
[x]	[ ] Clinical data
[x]	[ ] Dual use research of concern

Methods

n/a	Involved in the study
[x]	[ ] ChIP-seq
[x]	[ ] Flow cytometry
[x]	[ ] MRI-based neuroimaging
